# Towards the identification of the loci of adaptive evolution

**DOI:** 10.1111/2041-210X.12324

**Published:** 2015-02-12

**Authors:** Carolina Pardo-Diaz, Camilo Salazar, Chris D Jiggins

**Affiliations:** 1 Biology Program, Faculty of Natural Sciences and Mathematics, Universidad del RosarioCarrera 24 No 63C-69, Bogotá 111221, Colombia; 2 Department of Zoology, University of CambridgeDowning Street, Cambridge, CB2 3EJ, UK

**Keywords:** adaptation, candidate genes, DNA mapping, expression, functional analysis, QTL, resequencing

## Abstract

**1.** Establishing the genetic and molecular basis underlying adaptive traits is one of the major goals of evolutionary geneticists in order to understand the connection between genotype and phenotype and elucidate the mechanisms of evolutionary change. Despite considerable effort to address this question, there remain relatively few systems in which the genes shaping adaptations have been identified.

**2.** Here, we review the experimental tools that have been applied to document the molecular basis underlying evolution in several natural systems, in order to highlight their benefits, limitations and suitability. In most cases, a combination of DNA, RNA and functional methodologies with field experiments will be needed to uncover the genes and mechanisms shaping adaptation in nature.

Understanding how diversity arises and is maintained in nature has been a major question in evolutionary biology since ancient times (Lewontin 1974; Mayr 1982). Until recently, addressing this question was methodologically limited, but the recent and increasing development of molecular and genomic tools has now equipped us better to face the challenge. Consequently, much of modern research in evolution is devoted to identifying the genes shaping adaptive phenotypes (Hoekstra & Coyne 2007; Stern & Orgogozo 2009; Nadeau & Jiggins 2010; Jones *et al*. 2012; Kunte *et al*. 2014). This has and will continue to contribute to answering important questions about how evolution proceeds: Do adaptations arise gradually through many small mutations, via large leaps of major effect or both? Is the evolution of similar traits in different lineages the product of mutations in the same genes? Are particular kinds of adaptive mutations more likely than others, such as gene regulatory or protein-coding mutations? Here, we will focus on the methods used to address these questions.

The narrowing of the genomic regions controlling adaptations has employed mainly three different approaches termed *forward genetics*, *reverse genetics* and *candidate gene* (Stinchcombe & Hoekstra 2007; Nadeau & Jiggins 2010). *Forward genetics* approaches seek to identify genes underlying a known adaptive trait (Stinchcombe & Hoekstra 2007; Nadeau & Jiggins 2010; Stapley *et al*. 2010), while *reverse genetics* approaches refer to the detection of selection signatures across the genome without necessarily have a prior knowledge of the associated phenotype (Stinchcombe & Hoekstra 2007; Bonin 2008; Ellegren & Sheldon 2008; Stapley *et al*. 2010). Both *forward* and *reverse* genetics approaches are currently benefiting from the recent advances in sequencing technology, although its application still faces several challenges related to data storage, data analysis and cost that, although decreasing, can be challenging especially for organisms with large genomes (Wang, Gerstein & Snyder 2009).

Alternatively, the *candidate gene* approach relies on existing knowledge about the genes participating in the formation of the adaptive phenotype under investigation in other organisms; a correlation between trait variation and allelic polymorphism suggests the use of the candidate gene in shaping the adaptive trait (Luikart *et al*. 2003; Haag *et al*. 2005; Hoekstra *et al*. 2006; Mundy 2009). Any of the above approaches, alone or often combined, can narrow a strong set of candidate genes underlying adaptations. A further validation of these candidates can benefit from the exploration of the gene expression patterns and the application of functional assays such as knockouts, knockdowns and/or transgenics. However, a relation between a candidate gene and a presumptive adaptive phenotype does not constitute the unequivocal detection of the ‘loci of evolution’. The definite connection between gene function and adaptation also requires the implementation of selection experiments that test the adaptive consequences of the individual alleles of these candidates (Fig.1). Unfortunately, this has been rarely achieved, perhaps due to the difficulty of replicating evolutionary processes under controlled conditions (Colosimo *et al*. 2005).

**Fig 1:**
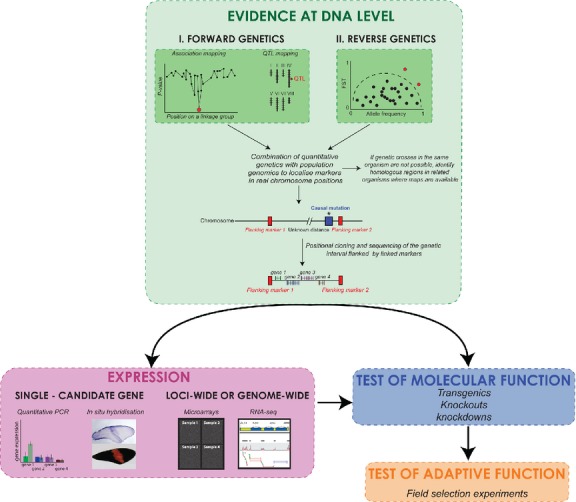
Methodological processes useful to identify the loci underlying adaptation. Ideally, phenotype–genotype association studies, followed by the profiling of gene expression, functional tests and selection tests should be combined to identify a gene(s) as involved in shaping an adaptive trait. Evidence at the DNA level was adapted and modified from (Stinchcombe & Hoekstra 2007; Barrett & Hoekstra 2011). *In situ* hybridization shows expression of the gene *optix* in wings of *Heliconius melpomene* (Photo: Bob Reed) (Reed *et al*. 2011). Photographs of microarray and RNA-seq by Carolina Pardo-Diaz.

## Narrowing down adaptations at the DNA level

### Forward genetics approaches

*Forward genetics* methods work towards finding the genetics controlling a known adaptive trait (i.e. one that is known to increase organismal fitness and/or reproduction in a particular context and where coefficients of selection have been measured and/or the response to changes in the selective agent has been quantified; see section IV: *assays of allele effects on fitness with field studies*). *Forward genetics* methods consist mainly of two different approaches: (i) association mapping using sexual populations that recombine in nature and (ii) quantitative trait loci (QTL) mapping using pedigrees, both of which survey a large number of molecular markers across the genome of individuals segregating for the adaptive trait of interest in order to identify gene regions as well as genes that are responsible for its variation (Stinchcombe & Hoekstra 2007; Nadeau & Jiggins 2010; Slate *et al*. 2010; Stapley *et al*. 2010; Martin & Jiggins 2013; Savolainen, Lascoux & Merila 2013; Wray 2013; Dittmar *et al*. 2014; Zuellig, Kenney & Sweigart 2014). Crucially in both cases, the methods involve identifying associations between the phenotypes of individuals with genetic variants. Thus, *forward genetics* is only useful where there is variation in a population for a particular trait or where such a variable population can be generated in the laboratory via crosses. This approach, however, is not useful to map differences in species that are completely isolated reproductively.

The association mapping approach, termed a ‘genome-wide association study’ (GWAS) when applied across the whole genome, takes advantage of the historical recombination in wild populations in order to detect non-random associations between genomic markers scattered across the genome and the adaptive trait of interest (Shimizu & Purugganan 2005; Stinchcombe & Hoekstra 2007; Hunter, Wright & Bomblies 2013). Using historical recombination increases the resolution in the detection of the locus (or loci) controlling the adaptive trait under study as the segregation would be much larger than that of a progeny of a experimental biparental population (Hwang *et al*. 2014). Thus, GWAS works by identifying non-random association of alleles between a locus with the adaptive trait (i.e. linkage disequilibrium, LD), as consequence of the action of natural selection (Long & Langley 1999; Shimizu & Purugganan 2005; Stranger, Stahl & Raj 2011). Until recently, GWAS applied genetic markers such as AFLPs, microsatellites and single copy gene markers to sample genetic variation (Table 1) (Holliday, Ritland & Aitken 2010; Holliday, Wang & Aitken 2012), but more recently, high-throughput sequencing approaches are used [such as reduced representation sequencing (Altshuler *et al*. 2000; Hohenlohe *et al*. 2010), low-coverage genotyping (Andolfatto *et al*. 2011; Elshire *et al*. 2011) and genome resequencing (Nielsen *et al*. 2011)]. Coupled with advances in bioinformatics, the use of GWAS to investigate the genetics of adaptation is increasingly popular (Hunter, Wright & Bomblies 2013). In lodgepole pines, for example, the use of restriction-site associated DNA sequencing (RAD-seq) generated markers for a GWAS of variation in serotiny (adaptation of cones to remain closed and retain seeds until a forest fire opens them) and revealed that at least 11 loci are involved in the natural variation of this trait (Parchman *et al*. 2012). Similarly, in *Arabidopsis thaliana,* a GWAS identified a *cis*-regulatory polymorphism at the *AtHKT1;1* locus as the factor controlling adaptive variation in leaf Na^+^ accumulation capacity (Baxter *et al*. 2010a). Additionally, several GWASs have identified loci with signals of human adaptations including genes involved in immunity, cancer, infection, reproduction, healing and height (Hancock *et al*. 2011b; Jarvis *et al*. 2012; Lachance *et al*. 2012; Scheinfeldt & Tishkoff 2013). The power of association-based methods to detect a true association between a SNP and an adaptive trait largely depends on the phenotypic variance of the population explained by the SNP. Such phenotypic variance is determined by how strongly the alternative allelic variants differ in their phenotypic effects (effect size) and their frequency in the sample (Korte & Farlow 2013). Association-based methods are therefore biased towards detecting large-effect loci, although this is probably true of all methods described here (Rockman 2011; Martin & Jiggins 2013). This bias can be reduced by using extremely large sample sizes to maximize the genetic variance within the sample (Bodmer & Bonilla 2008; Korte & Farlow 2013). However, in some cases GWAS in humans using large sample sizes have shown that a single phenotype may be controlled by many minor effect loci that may explain only a small proportion of the trait heritability, which has limited the identification of causal variants (Rockman 2011). This limitation of GWAS is highly relevant to the study of the genetics of adaptive traits with polygenic inheritance, which may be the majority of adaptive traits (Rockman 2011; Turchin *et al*. 2012).

**Table 1:** Examples of natural adaptations investigated at the molecular level, the methodological approaches followed and the genes identified (if they have)

Organism	Adaptive trait	DNA evidence	Expression Profiling	Functional test	Genes identified/suggested	References
*Arabidopsis thaliana*	Flowering time	Forward genetics approaches – association mapping and QTL mapping	Microarray	Complementation test	*FRI* and *FLC*	Aranzana *et al*. (2005), Lempe *et al*. (2005), Bergelson & Roux (2010), Salomé *et al*. (2011)
*Arabidopsis thaliana*	Pathogen resistance	Forward genetics approaches – association mapping and QTL mapping	RNA blot analysis	Mutagenesis	*Rpm1*	Grant *et al*. (1995), Tornero *et al*. (2002), de Torres *et al*. (2003), Aranzana *et al*. (2005)
*Gasterosteus aculeatus* (threespine stickleback)	Armour plate patterning	Forward genetics approaches – association mapping and QTL mapping	qRT-PCR	Transgenics	*EDA*	Colosimo *et al*. (2005), Jones *et al*. (2012)
*Gasterosteus aculeatus* (threespine stickleback)	Loss of pelvic spines	Forward genetics approaches – QTL mapping	qRT-PCR and *in situ* hybridization	Transgenics	*Pitx1*	Shapiro *et al*. (2004), Shapiro, Bell & Kingsley (2006), Chan *et al*. (2010)
*Peromyscus sp*.	Coat colour	Forward genetics approaches – QTL mapping and reverse genetics approaches	qRT-PCR, *in situ* hybridization and antibodies	Transgenics	*Agouti*	Manceau *et al*. (2011), Linnen *et al*. (2013)
Tetrapoda	Land colonization	Reverse genetics approaches – comparative genomics	RNA-seq, *in situ* hybridization	Transgenics	Several genes	Amemiya *et al*. (2013)
*Drosophila* sp.	Male wing pigmentation involved in courtship	Reverse genetics approaches – genotyping of candidate genes	Immunochemistry	Transgenics	*Yellow*	Gompel *et al*. (2005), Prud'homme *et al*. (2006)
	Spatial regulation of pigmentation genes involved in wing spot formation	NA	*In situ* hybridization, Western blot, immunochemistry, microarray, qRT-PCR	RNAi screen, mutants, transgenics	*Dll*	Arnoult *et al*. (2013)
*Thlaspi caerulescens*	Zinc accumulation	Forward genetics approaches – association mapping and QTL mapping	Microarray and Northern blot	Yeast complementation analysis	*MT2* and *MT3*	Assunção *et al*. (2006), Hassinen *et al*. (2007)
*Picea sitchensis* (sitka spruce conifer)	Wood physical attributes	Forward genetics approaches – association mapping	Microarray	NA	β-expansin, Tubulin 3B, Galactosyl-transferase	Holliday, Ritland & Aitken (2010), Beaulieu *et al*. (2011)
*Gasterosteus aculeatus* (threespine stickleback)	Gill pigmentation	Forward genetics approaches – QTL mapping	*In situ* hybridization and allele-specific expression	NA	*Kitlg*	Miller *et al*. (2007)
*Heliconius melpomene* and *Heliconis erato*	Wing colour pattern	Forward genetics approaches – QTL mapping	Microarray, *in situ* hybridization, qRT-PCR	NA	*Optix, WntA*	Jiggins *et al*. (2005), Baxter *et al*. (2008, 2010b), Counterman *et al*. (2010), Reed *et al*. (2011), Hines *et al*. (2012), Martin *et al*. (2012)
*Papilio polytes*	Wing mimicry	Forward genetics approaches – association mapping	Immunochemistry, qRT-PCR, RNA-seq	NA	*doublesex*	Kunte *et al*. (2014)
*Rattus norvegicus*	Warfarin resistance	Reverse genetics approaches – microsatellite	Semi-quantitative RT-PCR	NA	*Vitamin K epoxide reductase (VKOR)*	Kohn, Pelz & Wayne (2003), Lasseur *et al*. (2006)
Cichlids	Visual pigment diversification	Reverse genetics approaches – genotyping of candidate genes	qRT-PCR on candidate genes	NA	*Opsin* genes	Hofmann *et al*. (2009)
*Ovis aries* (Soay sheep)	Coat colour	Forward genetics approaches – association mapping	qRT-PCR	NA	*Tyrp1*	Beraldi *et al*. (2006), Gratten *et al*. (2007)
Vertebrates	Pigmentation	Reverse genetics approaches – genotyping of candidate genes	qRT-PCR	NA	*Mc1r*	Hoekstra & Nachman (2003), Nachman, Hoekstra & D’ Agostino (2003), Mundy *et al*. (2004), Rosenblum, Hoekstra & Nachman (2004), Hoekstra *et al*. (2006), Hubbard *et al*. (2010)
*Chrysomela aeneicollis* (montane beetle)	Adaptation to local thermal conditions	Reverse genetics approaches – genome-wide SNP typing	Western blot	NA	*Pgi – Hsp70*	Rank (1992), Dahlhoff & Rank (2000)
*Chaenocephalus aceratus* and *Pleuragramma antarcticum* (Antarctic notothenioid fishes)	Craniofacial skeletal morphology	Reverse genetics approaches – genotyping of candidate genes	*In situ* hybridization	NA	*col1a1, col2a1b* and *col10a1*	Albertson *et al*. (2010)
*Pseudopodoces humilis* (ground tit)	High altitude adaptations	Reverse genetics approaches – whole-genome sequencing	NA	NA	Adrenaline response and hormone biosynthesis genes	Cai *et al*. (2013)
*Chrysemys picta bellii* (western painted turtle)	Extreme anoxia and tissue freezing	Reverse genetics approaches – whole-genome sequencing	RNA-seq	NA	Tumour suppression genes, glucose transport genes and the miR-29b micro RNA	Bradley Shaffer *et al*. (2013)
Tapeworms	Parasitism adaptations	Reverse genetics approaches – whole-genome sequencing	RNA-seq	NA	Apomucin gene family, antigen B gene family, Hsp70 gene family	Tsai *et al*. (2013)
*Falco peregrinus* (peregrine falcon) and *Falco cherrug* (saker falcon)	Predation adaptations	Reverse genetics approaches – whole-genome sequencing	RNA-seq	NA	Olfactory receptor genes, beak development genes	Zhan *et al*. (2013)
*Pteropus alecto* (fruit bat) and *Myotis davidii* (insectivorous bat)	Flight and immune adaptations	Reverse genetics approaches – whole-genome sequencing	NA	NA	Repair of genetic damage genes, skin elasticity genes, muscle contraction genes, innate immunity genes	Zhang *et al*. (2013)
*Ambystoma mexicanum* (Mexican axolotl)	Paedomorphosis	Forward genetics approaches – QTL mapping	Microarray	NA	Thyroid hormone-response genes	Voss & Shaffer (1997), Page *et al*. (2008, 2009, 2010), Huggins *et al*. (2012)
Cichlid fish	Colour pattern	Forward genetics approaches – association mapping and QTL mapping	qRT-PCR	NA	*Pax7*	Streelman, Albertson & Kocher (2003), Roberts, Ser & Kocher (2009)
*Bicyclus anynana*	Eyespots	Genotyping of candidate genes	Immunohistochemistry	NA	Distal-less	Beldade, Brakefield & Long (2002)
*Rana chensinensis* and *R. kukunoris* (ranid frogs)	Adaptation to high elevation	NA	Transcriptomic analysis	NA	125 protein-coding genes	Yang *et al*. (2012)
*Ipomoea* sp.	Floral colour	Reverse genetics approaches – genotyping of candidate genes	*In situ* hybridization and Northern blot	Complementation test and enzyme assay	Genes in flavonoid biosynthesis	Durbin *et al*. (2003), Zufall & Rausher (2003, 2004)
*Coregonus* spp. (lake whitefish)	Growth, swimming activity, gill rakers and condition factor	Reverse genetics approaches – genome-wide SNP typing Forward genetics approaches – QTL mapping	Microarray	NA	Several genes	Rogers & Bernatchez (2005), Derome *et al*. (2008), Renaut *et al*. (2011)
*Littorina saxatilis*	Local adaptation	Reverse genetics approaches – AFLPs	ESTs genomic scan	NA	Two loci	Wood *et al*. (2008), Galindo, Grahame & Butlin (2010), Westram *et al*. (2014)
*Melitaea cinxia* (Glanville fritillary butterfly)	Dispersal rate and flight metabolism	Reverse genetics approaches – genotyping of candidate genes	NA	NA	*Pgi*	Haag *et al*. (2005)
*Anser indicus* and *Chloephaga melanoptera* (geese)	High altitude adaptation	Reverse genetics approaches – candidate genes	NA	Protein assay with same mutation in heterologous human protein	Haemoglobins	Jessen *et al*. (1991)
Chicken and Japanese Quail	Plumage colour	Reverse genetics approaches – candidate genes Forward genetics approaches – association mapping	Quantification of mRNA decay in mutant variant	NA	SLC45A2	Gunnarsson *et al*. (2007)
Lake Victoria cichlids	Light spectrum sensitivity – visual system	Reverse genetics approaches – candidate genes	NA	Protein assay	LWS	Terai *et al*. (2006)
Lakes Tanganyika and Malawi cichlids	Visual adaptation to deep-water habitats	Reverse genetics approaches–- genotyping of candidate genes	NA	Protein assay	RH1	Sugawara *et al*. (2005)
*Astyanax fasciatus* (Mexican cave tetra)	Albinism	Forward genetics approaches – QTL mapping	NA	Cell-based functional assay	*Oca2*	Protas *et al*. (2006)
*Ostrinia nubilalis* (European corn borer)	Sexual isolation pheromones	Forward genetics approaches–- association mapping	NA	NA	*Pher* and *Resp*	Dopman, Robbins & Seaman (2010)
*Cervus elaphus* (red deer)	Birth weight	Forward genetics approaches – association mapping and QTL mapping	NA	NA	One major QTL	Slate *et al*. (2002)
*Peromyscus* mice	Behavioural differences – burrow architecture	Forward genetics approaches – association mapping and QTL mapping	NA	NA	Four QTL	Weber, Peterson & Hoekstra (2013)
*Rana temporaria* (common frog)	Adaptation to altitude	Reverse genetics approaches – AFLPs	NA	NA	Eight outlier loci	Bonin *et al*. (2006)
*Pinus contorta (lodgepole pine)*	Cone serotiny	Reverse genetics approaches – genome-wide SNP typing	NA	NA	Eleven candidate loci	Parchman *et al*. (2012)
*Triticum* sp. (wheat)	Drought adaptation	Forward genetics approaches – association mapping	NA	NA	Several loci	Maccaferri *et al*. (2011)
*Phoxinus phoxinus* (cyprinid fish European minnow)	Body shape variation	Reverse genetics approaches – AFLPs	NA	NA	Several loci	Collin & Fumagalli (2011)
*Mimulus* sp.	Floral morphology	Forward genetics approaches – QTL mapping	NA	NA	Several loci	Bradshaw *et al*. (1998)
	Floral pigmentation	Reverse genetics approaches – candidate genes	qRT-PCR	Virus-induced gene silencing (VIGS)	R2R3-MYB	Streisfeld & Rausher (2009), Streisfeld, Young & Sobel (2013)
*Leptosiphon* sp.	Floral characters associated with the mating system	Forward genetics approaches – association mapping and QTL mapping	NA	NA	Several loci	Goodwillie, Ritland & Ritland (2006)
*Fundulus heteroclitus*	Adaptation to local water temperature	NA	*In vivo* reporter gene expression of candidate gene	Protein activity	Lactate dehydrogenase-B gene (*Ldh-B*)	Schulte *et al*. (2000)
*Drosophila yakuba* and *Drosophila santomea*	Body colouration	NA	Expression constructs with mutant versions (*in situ* hybridization)	Transgenics	*Tan* and *Yellow*	Jeong *et al*. (2008)

It is worth-noting that the suitability of a GWAS approach in discovering adaptation genes depends on additional factors that are highly dependent on the study system. For instance, the extent of genomic LD can introduce bias in a GWAS, so it is recommended to use high-density genotyping platforms that generate enough SNPs to examine the background level of LD in each population in order to differentiate LD outliers from genomic background (Alhaddad *et al*. 2013; Porto-Neto, Kijas & Reverter 2014). Also, obtaining the minimum number of markers necessary for a successful GWAS relies on factors such as genome size, number of individuals and number of groups or populations included, which all influence the genotyping effort required to get enough coverage and overlap between samples (Davey *et al*. 2011). Technical replicates with sufficient coverage may also be needed in order to assess the reproducibility of the genotyping technique and the quality of SNP calling, both of which can affect the proper calling of heterozygous sites and the reliability of GWAS (Hong *et al*. 2012; Nielsen *et al*. 2012).

QTL mapping is an alternative approach to understand the genetic basis of a known adaptive trait. It relies on the generation of mapping crosses to create a genetically variable and recombinant population in the laboratory and uses statistical analyses to correlate the quantitative variance of adaptive traits with molecular markers distributed across the genome and identify chromosomal regions contributing to phenotype differentiation (Ellegren & Sheldon 2008; Mackay, Stone & Ayroles 2009; Nadeau & Jiggins 2010; Stapley *et al*. 2010; Slate 2013) (Table 1). In sticklebacks, for example, pelvic structures and pigmentation play adaptive roles as defensive structures against predation and crypsis, respectively, and the genetic regions controlling natural adaptive variation at these two characters were both found by QTL mapping with microsatellites (Shapiro *et al*. 2004; Miller *et al*. 2007). In a similar way, QTL mapping using AFLPs revealed that only three genomic intervals modulate most of the observed adaptive wing colour variation in the butterfly *Heliconius erato* (Papa *et al*. 2013). A growing number of studies have applied next-generation sequencing techniques to QTL mapping. In the perennial ryegrass *Lolium perenne,* the identification of three QTL that explained nearly 40% of the resistance to stem rust was achieved using RAD-seq (Pfender *et al*. 2011). Similarly, the identification of a QTL involved in spinosad resistance in the diamondback moth *Plutella xylostella* applied a RAD-seq approach (Baxter *et al*. 2011).

Although QTL mapping has been historically useful in narrowing broad regions associated with traits of interest, their power relies on obtaining large families. Unfortunately, obtaining enough recombinant offspring is not always possible due to the nature of many organisms, and thus, the power and applicability of QTL may be limited. Also, this method faces some limitations related to estimating effect size, number of loci [or Quantitative Trait Nucleotides (QTNs)] and their interactions contributing to adaptation. First, the null hypothesis for QTL mapping is the absence of a QTL and not the presence of infinitesimal QTL, so it is not so applicable for the study of adaptive traits shaped by many minimal effect loci (Rockman 2011). In line with this argument, empirical data show that QTL mapping generally finds a skewed L-distribution of effect sizes (with few large-effect loci accounting for most of the variation). It is also widely known that if QTL mapping is performed with small sample sizes, the magnitude of the identified QTL is likely to be overestimated (‘Beavis effect’), the power of detecting medium to small effect QTL is limited, and the signature of several linked QTL may be blurred into a single large QTL (Mackay, Stone & Ayroles 2009; Nadeau & Jiggins 2010; Rockman 2011; Slate 2013). A recent review of QTL mapping studies showed that there is clear evidence for an upward bias in the magnitude of QTL estimates, particularly when sample sizes are small, but possibly even when they are as large as 1000 individuals (Slate 2013). Nonetheless, QTL mapping is often an important initial step towards gene discovery, which can lead to further genomic scans (such as GWAS) to establish whether QTL can be replicated (Slate 2013). This is exemplified by a study of horn morphology in Soay sheep, where a QTL was first finely mapped (Johnston *et al*. 2010) and then confirmed using an association study. This identified the gene *RXFP2* as the gene control variation in horn phenotype (Johnston *et al*. 2011).

### Reverse genetics approaches

*Reverse genetics* (or genome scan) approaches refer to the genome-wide sampling of loci in order to detect regions with footprints of selection and thus detect selective (adaptive) loci even without a prior knowledge of their associated phenotypic trait(s) (Luikart *et al*. 2003; Storz 2005; Martin & Jiggins 2013). This approach benefits from the fact that no experimental crosses are needed but instead, variation in natural populations can be used (Schlötterer 2003; Stinchcombe & Hoekstra 2007) and also, it should be less biased towards large-effect loci (as it identifies actual targets of selection) (Martin & Jiggins 2013).

The *reverse genetics* strategy involves a comprehensive sampling of independent loci across the entire genome and the application of statistical analyses to identify loci with genetic variation indicative of selection that are possibly involved in adaptation (Balding & Nichols 1995; Nicholson *et al*. 2002; Luikart *et al*. 2003; Vitalis *et al*. 2003; Joost *et al*. 2007; Foll & Gaggiotti 2008; Coop *et al*. 2010; Nunes *et al*. 2012; De Mita *et al*. 2013; Frichot *et al*. 2013; Martin & Jiggins 2013; de Villemereuil *et al*. 2014). These statistics can be applied both at intra- and inter-population level.

Within a single population, measures of genetic diversity and distribution of genetic polymorphism (or allele frequency spectrum, AFS) with statistics such as π and Tajima's D are expected to be influenced by selection fixing an advantageous allele. This fixation also leads to a reduction of genetic diversity in the surrounding sequences creating a selective sweep, due to genetic hitchhiking. The location of such selective sweeps can be detected by evaluating patterns of linkage disequilibrium (LD) (Kim & Nielsen 2004). The detection of hard sweeps (i.e. new mutations that arise and quickly go to fixation) is facilitated by the pattern of strong reduced nucleotide polymorphism at the selected locus and its neighbour regions (Pritchard, Pickrell & Coop 2010; Olson-Manning, Wagner & Mitchell-Olds 2012). In contrast, detecting the signal of soft sweeps (when the selected allele has been already segregating in the population before being swept) is more difficult because the selected haplotype may be impossible to differentiate from the genetic background (Pritchard, Pickrell & Coop 2010; Olson-Manning, Wagner & Mitchell-Olds 2012). Furthermore, the signal of a selective sweep may be lost fairly rapidly over time especially in large populations. According to simulation studies, this limitation can be overcome with the combination of LD scans with AFS scans (Pavlidis, Jensen & Stephan 2010). Nonetheless, false positives remain a problem. For example, Pavlidis *et al*. (2012) used simulated data under neutrality in *Drosophila* and still detected false positive sweeps with misleading biological functions (Pavlidis *et al*. 2012). Due to these constraints, genomic scans of selective sweeps are less commonly used as a first step in identifying adaptive loci, yet they are useful to confirm sweep signals at loci involved in adaptations (Hohenlohe *et al*. 2010; Nadeau *et al*. 2013).

Demographic factors can also affect nucleotide polymorphism and produce patterns of summary statistics (like π and Tajima's D) easily confused with those expected by the action of selection (Stinchcombe & Hoekstra 2007). However, it is expected that demography affects the genome as a whole. The problem can therefore be ameliorated to some degree by using genome-wide data to evaluate the demographic history of the species and provide a neutral expectation against which particular regions can be tested for the influence of selection (McVicker *et al*. 2009; Pool *et al*. 2010; Li & Durbin 2011). Selective sweeps also influence haplotype structure, and inferences based on more complex measures, not based on SNPs alone, are likely to be more powerful (Gusev *et al*. 2009, 2011; Pavlidis *et al*. 2013).

Inter-population measures can also be used to detect footprints of selection across the genome. The most common approach is to apply tests based on *F*_ST_ to reveal loci where the fixation of alternative alleles in each population results in greater differences in allele frequency than expected under neutral evolution (Beaumont & Nichols 1996; Bonhomme *et al*. 2010; Narum & Hess 2011; Martin & Jiggins 2013; de Villemereuil *et al*. 2014; Lotterhos & Whitlock 2014). Alternatively, there are methods to detect selection based on correlations of genetic data with environmental changes (Joost *et al*. 2007; Coop *et al*. 2010; De Mita *et al*. 2013). Simulation studies under a monogenic scenario have shown that *F*_ST_-based methods are able to consider complex population history and structure (when known) and are less prone to detect false positives; however, they fail to detect true selection outliers when selection is not strong and their results are strongly dependent on the demographic model implemented (De Mita *et al*. 2013; Lotterhos & Whitlock 2014). Additionally, it has been recently pointed out that the use of relative measures in detection of outliers (e.g. *F*_ST_) may be misleading when searching regions in the genome involved in adaptation and speciation (Noor & Bennett 2010; Martin *et al*. 2013; Cruickshank & Hahn 2014). In some genomic regions, for example, genetic divergence between species of recent origin may lead to a decrease in genetic variability and recombination rate as consequence of the speciation process, which can be misinterpreted as signatures of positive or negative selection acting within species. Similarly, regions of the genome with restricted gene flow compared to the genomic background (measured with *F*_ST_) are usually interpreted as islands of divergence (Turner, Hahn & Nuzhdin 2005; Ellegren *et al*. 2012). However, this signal also may be the result of positive or background selection that acted, in the past, on the ancestral population (Noor & Bennett 2010; Cruickshank & Hahn 2014). Thus, the misinterpretation of *F*_ST_ patterns can confuse processes of adaptation with those of speciation. It has been suggested that absolute measures of genetic divergence (that do not rely on allelic frequencies), such as D_XY_ distance, may be better, but these also fail to detect some regions known to be under selection (Cruickshank & Hahn 2014). A related factor to consider is the relationship between recombination and selection. Divergence and low diversity is commonly higher in regions with low recombination (e.g. centromeres), which may result from natural selection or just be a consequence of limited recombination (Turner & Hahn 2010). There may also be a positive relationship between recombination and mutation, which would increase the variation available for selection to act upon (Cutter & Payseur 2013). However, to date, studies are still needed that investigate how recombination rate varies across the genome and in candidate regions for adaptation and speciation.

Environment–genetic correlation methods, on the other hand, are more powerful and work well under different strengths of selection. On the other hand, they may have a high false discovery rate (FDR) if genetic correlations between populations are not accounted for (De Mita *et al*. 2013). In some cases, correct estimation of the environmental variables can be challenging, potentially limiting application of this latter method. In polygenic scenarios, where multiple loci of small effect underlie a single trait, both environment–genetic correlation methods and *F*_ST_-based methods show lower power of detection compared to major locus genetic architecture (de Villemereuil *et al*. 2014). Demographic effects (such as a high level of hierarchical population structure) and its correlation with an environmental variable underlying the selective pressure also affect the power of detection in these polygenic cases (de Villemereuil *et al*. 2014).

Despite the limitation of environment–genetic correlation methods and *F*_ST_-based methods, both have helped in identifying outlier loci associated with adaptations in natural populations. Examples include the identification of whole-genome outlier SNPs associated with climatic adaptations in *Arabidopsis thaliana* (Hancock *et al*. 2011a), transcriptome-derived SNPs correlated with stress tolerance in coral reefs (Lundgren *et al*. 2013), outlier AFLP loci correlated with insecticide resistance in mosquitoes (Paris & Despres 2012), SNPs associated with adaptation to coastal environments in *Senecio lautus* (Roda *et al*. 2013) and SNPs associated with climatic adaptations in humans (Hancock *et al*. 2011b). A combination of population genetics and environmental correlations can help to reduce the number of false positives (de Villemereuil *et al*. 2014). This has been rarely done, although one exception is the identification of loci involved in host plant use in the large pine weevil *Hylobius abietis* (Manel, Conord & Despres 2009). Yet, ‘new more general and robust likelihood test are needed that are flexible enough to accommodate departures from classical demographic models’ (de Villemereuil *et al*. 2014).

Regardless of the method used, it is fundamental to validate that outliers are genuinely implicated in adaptation (Luikart *et al*. 2003; Barrett & Hoekstra 2011). This can be achieved by combining population genetics both within and between populations, and/or complementing *forward genetics* with *reverse genetics* (Stinchcombe & Hoekstra 2007; Butlin 2010; Hohenlohe, Phillips & Cresko 2010). The latter combination has been possible in wild populations of threespine sticklebacks where the confident identification of the *Eda* gene, responsible for the adaptive reduction of armour plates, was achieved by combining QTL mapping with SNP typing in wild populations (Colosimo *et al*. 2005) and also in *Heliconius* butterflies where the identification of the transcription factor *optix* controlling red adaptive wing pattern variation was possible thanks to the application of AFLP mapping (Baxter *et al*. 2008) followed by population genetic analyses on the focal region (Baxter *et al*. 2010b; Counterman *et al*. 2010; Reed *et al*. 2011).

### Candidate genes

The knowledge of candidate genes derived from other organisms can be combined with these approaches to identify genes that are associated with a particular adaptive phenotype in a different species (Table 1). Thus, either in crosses or in naturally varying phenotypes, candidate genes can be examined for evidence of their association with the trait of interest (Shimizu & Purugganan 2005; Stinchcombe & Hoekstra 2007). In this way, the variation in plumage colour in natural populations of the flycatcher *Monarcha castaneiventris* was shown to be associated with a single mutation of the previously identified pigmentation gene melanocortin-1 receptor (*MC1R*) (Mundy 2005; Uy *et al*. 2009). It is important to be selective at the time of applying the candidate gene approach. If there are many candidate genes associated with a phenotype, this might not be as fruitful as when there is a handful of strong candidates (Luikart *et al*. 2003). Furthermore, a major drawback of a candidate gene approach is that the literature becomes biased towards a few well-known candidate genes, which may not therefore be representative of their actual importance in evolutionary change (Mundy 2005). In addition, this approach makes the assumption that the genes that matter for evolution are necessarily few and of large effect.

As genomic technologies become more widely available and more information on gene interactions and pathways exists, researchers are moving away from a simplistic candidate gene approach and are applying larger studies that evaluate, at once, the role of multiple genes in a pathway suspected to affect the formation of the adaptive trait (candidate pathway approach) (Suh & Vijg 2005). For example, the flavonoid pathway has been a model system in plants that has helped understanding the genetics underlying flower coloration and other evolutionary processes, including the role of gene duplication in the evolution of novel phenotypes (Des Marais & Rausher 2008), causes of evolutionary rate variation among genes (Lu & Rausher 2003) and the relative importance of coding vs. regulatory mutations in the evolution of ecologically relevant traits (Wessinger & Rausher 2012). Flower coloration is an adaptive trait (Kopp 2009) caused by anthocyanin pigments, whose production requires at least six sequential reactions catalysed by six different enzymes in the anthocyanin pathway (Rausher 2006). The pathway candidate approach in flowers has not only led to identification of the particular enzymes involved in synthesizing red/orange, blue/magenta and blue/purple pigments (Zufall & Rausher 2003; Rausher 2006) but also helped in the identification of the transcriptional complex, composed by bHLH and MYB domain transcription factors, responsible for natural variation in flower coloration among many plant species (Rausher 2006; Kopp 2009). Despite the potential of the candidate pathway analysis in the study of natural adaptation, to date, it is poorly applied to evolutionary studies and largely remains restricted to studies on the genetics of human diseases (Suh & Vijg 2005).

### Characterizing regions narrowed by forward or reverse genetics

Outlier loci have been identified in a wide range of species (Bonin *et al*. 2006; Minder & Widmer 2008; Apple *et al*. 2010), but fewer studies have moved from their detection to the characterization of underlying QTNS, genes or networks controlling adaptation (Minder & Widmer 2008; Wood *et al*. 2008; Paris *et al*. 2010; Midamegbe *et al*. 2011; Rockman 2011; Kunte *et al*. 2014). Following up on a particular outlier locus can be time-consuming and technically demanding, but will be a necessary step in order to find the genes or regulatory elements involved in adaptation. One of the most popular approaches is a library-based search of genomic regions flanked by outliers, followed by positional cloning using BACs and sequencing of the genetic interval (Butlin 2010; Nunes *et al*. 2012). This has been successfully applied in the identification of several wing colour pattern loci in *Heliconius* butterflies (Baxter *et al*. 2010b; Counterman *et al*. 2010), in *Papilio polytes* to find mimicry ‘supergenes’ (Kunte *et al*. 2014) and, in the marine gastropod *Littorina saxatilis*, it has been used to pinpoint candidate loci for local adaptation (Wood *et al*. 2008).

Pathway analysis (where multiple ‘outlier’ SNPs are analysed jointly) also offers an interesting, yet underutilized, tool to discover gene sets likely involved in the formation of a trait of interest (Pan *et al*. 2014). This is because although the detection of multiple outlier SNPs associated with a trait (with GWAS, for example) offers an insight into its underlying genetics, this alone may not be very informative in the case of quantitative polygenic traits, because individual SNPs only account for a small part of the trait variance (Mokry *et al*. 2013). However, pathway analysis is challenging, because a large number of SNPs per individual need to be considered in predictive models. Machine learning methods such as multi-dimensional reduction (MDR), support vector machines (SVM), neural networks (NN) and random forest (RF) are capable of dealing with this dimensionality problem in a flexible manner and can effectively select important variables from irrelevant ones (Goldstein *et al*. 2010; Gonzalez-Recio & Forni 2011; González-Recio, Rosa & Gianola 2014; Yang & Charles Gu 2014). In particular, RF analysis has been particularly useful in pathway analysis because interactions are implicitly modelled (De Lobel *et al*. 2010; Chung & Chen 2012) and it is straightforward to understand and interpret (Goldstein *et al*. 2010; González-Recio, Rosa & Gianola 2014). Nonetheless, this methodology has been primarily applied to the study of complex diseases in humans, considering a small number of SNPs (Chang *et al*. 2008; Ballard *et al*. 2010). Its application to large SNP data sets (such as those produced by next-generation techniques) is more complicated and requires the modification of certain standard assumptions (Goldstein *et al*. 2010; Chen & Ishwaran 2012). Still, this methodology offers an interesting option to the study of genetic pathways shaping natural adaptations. Pioneering work on this includes the search for loci involved in environmental adaptation in *Senecio lautus*. By comparing genomes of phenotypically contrasting parapatric populations, researchers assessed genetic association at different levels, from SNPs to physiological pathways (Roda *et al*. 2013).

Alternatively, when ‘outlier’ markers associated with a trait have been identified, they can be mapped back to a reference genome or to available linkage maps (in the same species or a closely related one), to detect whether they fall in or near protein-coding genes possibly affecting the trait. For example, in the rainbow and steelhead trout, species where no reference genome exists but linkage maps do, GWAS coupled with SNP mapping discovered that migration in these species has a complex quantitative genetic basis, resulting from many loci of small effect (Hecht *et al*. 2013). A similar approach was used to study the genetics of the adaptive natural variation in female abdominal pigmentation in *Drosophila melanogaster* and determined that variation in this trait is under the control of *cis*-regulatory regions of the genes *tan* and *bric-à-brac* (Bastide *et al*. 2013). With the increasing availability of whole-genome sequences in a wide variety of taxa and the possibility to develop genomic resources at a reasonable cost in species that lack them, it is becoming more feasible to apply this strategy in non-model species.

## Gene expression profiling

When DNA variation associated with phenotypic change does not occur in protein-coding regions, examining the expression pattern of genes provides an important complementary method to test for regulatory change (Rockman & Kruglyak 2006; Hoekstra & Coyne 2007; Hofmann *et al*. 2009). Although the detection of one or more differentially expressed genes provides information about potential candidates involved in the production of the trait of interest, this does not necessarily imply that all (or any) of them bear the causal variants. Differential expression may result from gene regulation due to upstream mutation(s), which may lie elsewhere in the genome (Stern & Orgogozo 2008; Kopp 2009; Stern & Orgogozo 2009). Thus, a combination of DNA data with expression data is often needed to determine whether the trait has a *cis-*regulatory basis (i.e. when differential expression and DNA polymorphism associated with phenotype both reside in the same locus) or it is *trans-*regulated. To this end, allele-specific expression (ASE) assays testing single or multiple genes offer a useful alternative to uncover the respective contributions of *cis*- and *trans*-regulatory variation (Knight 2004; Gilad, Rifkin & Pritchard 2008; Main *et al*. 2009; Wittkopp 2011).

Studies of gene expression can be conducted at either individual candidate loci (e.g. *in situ* hybridization, reverse-transcriptase quantitative PCR, immunochemistry) or many loci at once (e.g. microarrays, RNA-seq) depending on the information and resources available (Pavey *et al*. 2010). Studying thousands of transcripts allows a detailed and unbiased description of the genes involved in shaping natural evolution, and has the potential to identify entire genetic and developmental pathways driving adaptive variation. Thus, transcriptomic approaches can catalyse the discovery of multiple components of these gene networks, from the genes regulating phenotypic ‘switches’ to the repertoire of molecules responding to such genes of major effect using, for example, the recently developed Weighted Gene Co-Expression Network Analysis (WGCNA). This method offers the possibility to identify networks of co-expressed genes participating in the formation of a trait while at the same time points to candidate nodal ‘key’ genes likely controlling phenotypic variation only using gene expression data (Oldham, Horvath & Geschwind 2006; Filteau *et al*. 2013). This approach, however, does not identify the actual mutations controlling trait variance, and therefore, complementary DNA characterization on these candidates should also be carried out.

Microarrays have pioneered the genome-wide characterization of transcripts associated with adaptive natural variation. Almost a decade ago, microarrays were used in one of the first attempts to identify genes controlling beak morphology variation in Darwin's finches and showed that the calmodulin (CaM)-dependent pathway is a key component of the evolution of beak variation in these birds (Abzhanov *et al*. 2006). Similarly, microarrays showed that in limnetic Coregonine fishes, the parallel phenotypic evolution towards using the same ecological niche involves similar changes in expression at the same genes (Derome & Bernatchez 2006). Nonetheless, microarrays suffer from several limitations. Background levels of hybridization, differences in hybridization properties among probes and the restriction of interrogating only the transcripts included in the array are among the most common problems of this technique (Marioni *et al*. 2008).

Nowadays, high-throughput mRNA sequencing technology (RNA-seq) has the potential to overcome some of these limitations (Marioni *et al*. 2008; Wang, Gerstein & Snyder 2009). By using next-generation sequencing technologies, RNA-seq allows for a direct estimation of relative transcript abundance across the entire genome (Cheviron & Brumfield 2012) keeping the background noise low where sequences can be unambiguously mapped to unique regions of the genome (Marioni *et al*. 2008; Wang, Gerstein & Snyder 2009). This technique is not limited to detecting transcripts in organisms with a reference genome as the same RNA-seq data can be used to create a transcriptome assembly which is then used as a reference for read mapping (Grabherr *et al*. 2011), thus being particularly attractive for non-model organisms. This approach has recently been applied to naturally varying organisms such as the intertidal copepod *Tigriopus californicus*, where differences in thermal tolerance were associated with differential expression of heat-shock proteins (*Hsp*) and genes involved in ubiquitination and proteolysis (Schoville *et al*. 2012) and, in eucalyptus, RNA-seq has provided insights into the molecular mechanisms underlying the adaptation to water shortage (Villar *et al*. 2011). RNA-seq, as any other next-generation sequencing technique, presents limitations in terms of data storage, analysis and cost. Nonetheless, with RNA-seq, it is especially important to consider sequence coverage (directly related to the sequencing cost) because in organisms with large genomes and complex transcriptomes, more sequencing depth will be required for an adequate coverage (Wang, Gerstein & Snyder 2009) and/or where multiple replicates and comparisons will be needed to correctly tackle a particular trait.

Alternatively, when strong single candidate genes have been isolated at the DNA level or have been derived from transcriptomics studies, their further and complementary characterization can be done using reverse-transcriptase quantitative PCR (RT-qPCR). Several studies have applied RT-qPCR to profile transcription levels of particular candidate loci. For example, measuring the expression levels of the gene *Agouti* with RT-qPCR (implicated in producing pheomelanin in *Mus musculus*) demonstrated that *cis*-regulatory evolution at this gene was involved in adaptive variation in cryptic colouration of deer mice (Linnen *et al*. 2009). Similarly, adaptive differential retinal sensitivity in African cichlids inhabiting clear vs. turbid water relates to differences in *opsin* gene expression measured with RT-qPCR (Hofmann *et al*. 2009). However, RT-qPCR depends on the performance and specificity of primers so only the gene of interest is quantified (Busk 2014), making optimization a time-consuming process. Also, its accuracy is strongly reliant on the use of multiple control genes (e.g. reference or housekeeping genes) for normalization and correction of the multiple variation sources. A correct choice of control genes is not a trivial task. It is desirable to use more than one, as a single control gene can lead to normalization biases. Also, it is necessary to be sure that they are equally expressed across all the tissue types and species interrogated, as it can affect the accuracy of the calculation of relative expression differences between samples (Fedrigo *et al*. 2010).

The spatial distribution of expression patterns of candidate genes can be visualized using *in situ* hybridization (ISH), and this approach has provided the foundation for much of the field of ‘evo-devo’. The technique involves hybridizing an antisense RNA probe to an mRNA transcript, and it is a powerful method to characterize gene expression in tissues. The ISH procedure follows five major steps: (i) sample preparation, including fixation, mounting and ISH pre-treatment, (ii) probe preparation, (iii) hybridization, (iv) probe removal and (v) detection (Apostolopoulos 2001). Several studies have exemplified the usefulness of this technique to characterize the loci of adaptation. Shapiro *et al*. (2004) used ISH to compare profiles of expression of the gene *Pitx1* between benthic and marine sticklebacks and thus showed that a *cis*-regulatory element of *Pitx1* is responsible for pelvic size variation in fishes of the two environments (Shapiro *et al*. 2004). In the Darwin's finches, ISH patterns of expression of the genes TGFβIIr, β-catenin and Dickkopf-3 are differentially expressed in the developing pre-maxillary bone of embryos of species with different beak shapes, a trait associated with the exploitation of multiple ecological niches (Mallarino *et al*. 2011). In a similar way, ISH showed that in *Heliconius* butterflies, *cis*-regulatory evolution of the transcription factor *optix* drives the convergent evolution of red wing patterns in distantly related species (Reed *et al*. 2011). Nonetheless, ISH protocols are not easy to establish because the technique can be challenging to optimize (Abzhanov *et al*. 2008). Furthermore, it requires enough supply of organismal tissue at different developmental stages. This therefore requires raising a large number of individuals in a controlled environment or sampling enough individuals in the wild, specifically at the developmental points required (Abzhanov *et al*. 2008), which already imposes a limitation for many natural systems.

The use of immunohistochemistry provides an alternative to spatially localize the products of gene expression, and it is a much more forgiving technique than ISH (Abzhanov *et al*. 2008). It has been successfully applied to document the genetics of adaptive wing radiation in *Heliconius* (Martin *et al*. 2014) and the genes controlling male wing pigmentation in *Drosophila* (Gompel *et al*. 2005; Prud'homme *et al*. 2006). However, immunohistochemistry depends on the development of a species-specific antibody targeting the protein of interest, which can be time-consuming and expensive, or the availability of a cross-reactive antibody in a different species (Abzhanov *et al*. 2008). Just as ISH, immunohistochemistry also requires enough supply of organismal tissue at specific developmental points, limiting its application in many natural organisms.

Nonetheless, care should be taken in design and interpretation of gene expression assays. Variation in ecologically relevant traits is sometimes due to phenotypic plasticity (Hoffman & Goodisman 2007; Bossdorf, Richards & Pigliucci 2008; Whiteman & Agrawal 2009) which can be confounded with adaptive heritable variation. In this way, if the adaptive relevance of a trait has not been experimentally tested and it turns out to be a plastic phenotype, comparing gene expression in different conditions will yield a set of genes that do not contain the causal adaptive variants. Of course this is not to deny the importance of phenotypic plasticity in adaptation, but this is a subject beyond the scope of this paper (Ghalambor *et al*. 2007; Bossdorf, Richards & Pigliucci 2008; Hughes 2012).

## Assays of molecular function

The implementation of assays of molecular function such as transgenics, knockouts, knockdowns (with RNA interference (RNAi), for example) and gene replacement constitutes an important test to prove that a gene actually underlies natural variation (Shimizu & Purugganan 2005; Hoekstra & Coyne 2007; Pavey *et al*. 2012) and establish whether it is required and/or sufficient for the development of the adaptive trait (Abzhanov *et al*. 2008). Functional tests of candidate genes are well implemented in model organisms like *Drosophila,* yeast, nematodes and mice (Feder & Mitchell-Olds 2003; Heffer & Pick 2013) and provide the standard evidence for the genetic basis of trait variation. In these organisms, for example, the application of functional tests has confirmed the identification of the genes, and even mutations, controlling adaptive natural variation. In *Drosophila,* the application of a set of transgenic reporter assays found the actual mutations in the regulatory elements of the *ebony* gene controlling adaptive abdominal pigmentation in African natural populations (Rebeiz *et al*. 2009). Similarly, in the deer mouse *Peromyscus maniculatus*, the generation of *Agouti* knockouts confirmed the involvement of this gene in adaptive melanism (Kingsley *et al*. 2009).

Over the past several years, new functional protocols have been developed for a wide range of emerging organisms including *Daphnia,* wasps, crickets, ladybirds, cavefish and sticklebacks (Osanai-Futahashi *et al*. 2012; Pavey *et al*. 2012) and, although this is a field under active development, these experiments are still not feasible in all organisms and therefore impose a limitation in several non-model systems. Nonetheless, when functional assays are impossible in the target organism, it is still possible to use closely related species as ‘model’ organisms. For example, use of the retroviral vector RCAS in the chicken embryo implicated the genes *CaM, TGF*β*IIr,* β*-catenin* and *Dkk3* in controlling beak development which may imply a role in the evolution of Darwin's finches (Abzhanov *et al*. 2006; Mallarino *et al*. 2011). However, it is important to bear in mind that such experiments in ‘model’ species do not directly identify the role of natural variants. Similarly, in the sticklebacks, a functional test of the *Ectodysplasin-A* (*EDA*) gene using transgenics showed how this gene controls adaptive plate variation in natural populations (Colosimo *et al*. 2005), but the transgenic construct carried a mouse *EDA-A1* cDNA rather than the native stickleback ‘complete’ *EDA* allele. Although changes in plate phenotype were indeed observed, the results were variable. Three out of fourteen transgenic ‘low-plated’ fishes developed extra plates on their sides, but not in a consistent manner; the number and type of extra plates developed varied between and within individuals (different in each side) (Colosimo *et al*. 2005). In the future, it is hoped that experiments can be developed that more directly replicate the role of naturally occurring variants in their native species.

With the constant development of functional tools that were previously only available in more traditional ‘model’ organisms, now it is possible not only to pinpoint genes and mutations shaping natural adaptations but also to establish new organisms in which to study the genetics underlying evolution. Nonetheless, the task of developing more functional assays applicable to a wider range of organism is still needed. RNA interference (RNAi), for example, is a method for knocking down expression of a target gene and appeared to be easily accessible. However, in some taxa such as the Lepidoptera, it has proved to be highly problematic (Terenius *et al*. 2011). Responding to this need, recently developed approaches commonly referred as ‘genome editing tools’ and based on the use of engineered nucleases coupled to DNA recognition domains have been developed; these include zinc-finger nucleases (ZFNs), transcription activator-like effector nucleases (TALENs) and the clustered regulatory interspaced short palindromic repeats (CRISPR)/Cas9 endonuclease system (Gaj, Gersbach & Barbas Iii 2013; Wei *et al*. 2013). In all these ‘genome editing tools’, the DNA-binding module recognizes and binds the target gene while the nuclease module induces DNA double-strand breaks (Bassett *et al*. 2013; Gaj, Gersbach & Barbas Iii 2013; Wei *et al*. 2013). This activates either error-prone non-homologous end joining, which commonly introduces indels or frameshifts leading to the knockout of gene function, or homology directed repair, which allows the introduction of changes from single nucleotide changes to entire transgenes (Gaj, Gersbach & Barbas Iii 2013). These emerging technologies have already started to expand the ability to manipulate genes in less traditional organisms. For example, a related technique using zinc-finger nucleases (ZFNs) has allowed the mutation of genes related to the circadian clockwork in the monarch butterfly (*Danaus plexippus*) (Merlin *et al*. 2012). Interestingly, the (CRISPR)/Cas9 system is now viewed as a more attractive technical choice as their DNA recognition potential is bigger than that of ZFNs or TALENs, although given its recent development, at the moment it only has been applied to ‘model’ organisms (Bassett *et al*. 2013; Chang *et al*. 2013; Gaj, Gersbach & Barbas Iii 2013; Wei *et al*. 2013). Yet, the potential of (CRISPR)/Cas9 in the study of the genetics of adaptation in natural systems is very promising since it could be combined with classical genetic approaches (such as genetic complementation) to simultaneously map natural variation and functionally test the genes harbouring causal alleles (Turner 2014). This great advance in genome editing technologies opens new opportunities to decipher and test the molecular underpinnings of adaptations in a wider range of organisms and exemplifies how active research and development of tools broadens our methodological possibilities to answer longstanding questions.

In the future, transgenic tests should ideally involve replacement of alternate natural alleles at a locus, in order to demonstrate the functional value of particular substitutions. To date, this has only rarely, if ever, been achieved. Methods that involve knockouts or experiments involving ‘model’ species should be seen as a complement to other DNA or RNA approaches narrowing candidate genes likely shaping adaptive variation, but not a definitive test of adaptive function.

## Assays of ecological function

Although all the methods described above help to identify genes (or QTNs) contributing to adaptive phenotypic variation, it is crucial to perform field experiments either in nature or in conditions that closely mimic naturally occurring events, to evaluate that the trait of interest is indeed adaptive and, also, to test the fitness consequences of allelic substitutions at the causal genes (Barrett & Hoekstra 2011). Experimental evolution studies that link genes, phenotype and fitness have been possible under laboratory conditions and using organisms such as virus, bacteria and yeast, with very short generation times and where the replicated sequencing of whole genomes is feasible (Rokyta *et al*. 2005; Barrick *et al*. 2009; Araya *et al*. 2010; Brockhurst, Colegrave & Rozen 2011; Barrick & Lenski 2013). However, potential confounding effects or artefacts in those artificial systems make it hard to extrapolate the patterns and conclusions derived from them to a context of natural adaptation, highlighting the need to perform this experiments in natural systems (Barrett & Hoekstra 2011).

The most common approach to evaluate whether a trait has a direct impact on fitness in nature is by testing cause-and-effect relationships in a planned field experiment comparing different conditions (i.e. varying the suspected natural selection agent). There, phenotypic variation in the trait driven by differences in environment should be observed. Field experiments to test the adaptive value of traits in the wild come in a great variety and complexity of forms including *Q*_ST_–*F*_ST_ comparison (Leinonen *et al*. 2013), reciprocal transplants of hybrid individuals (Lowry *et al*. 2009), controlled introduction of live organisms to new environments (Reznick *et al*. 1997; Kapan 2001; Barrett & Schluter 2008; Irschick & Reznick 2009; Gompert *et al*. 2014) and replacing of the real organisms with synthetic replicas in nature primarily to quantify the impact of predation (Irschick & Reznick 2009; Merrill *et al*. 2012; Linnen *et al*. 2013). For example, Linnen *et al*. (2013) used plasticine mice models of two different colours in the field and found that light-coloured models matching light-coloured soil were less attacked by visually hunting predators (Linnen *et al*. 2013). Similarly, when *Anolis* lizards were introduced from the mainland into a series of islands, the introduced population evolved a different hind limb phenotype potentially as an adaption to the use of narrow surfaces (Losos, Warheitt & Schoener 1997). While these examples show that a trait affects organismal fitness in the wild, they do not tell us about how the genetic variation in the genes shaping those adaptive traits evolves in response to the experimental treatments, and thus, no connection between genotype, phenotype and fitness can be established.

In addition, QTL mapping has been useful to identify regions associated with habitat adaptation using reciprocal transplants of hybrid individuals [F2, backcross, recombinant inbred lines (RILs), near isogenic lines (NILs)] in contrasting environments (Bradshaw & Schemske 2003; Lowry *et al*. 2009). This approach allows evaluating both genotype–environment interactions and the effects of epistasis in fitness (Barrett & Hoekstra 2011).

To overcome this, when the identity of the gene underlying the formation of the adaptive trait is known (using the methods described above), one should study whether genetic variation in the causal gene evolves in the expected direction in response to differential treatments and also presents signatures of selection (Weinig *et al*. 2003). For instance, hybrid sticklebacks between a lake and a river population were transplanted into river and lake environments that differ in their parasitic diversity. Under the hypothesis that in order to survive and reproduce a host should resist to local parasites and pathogens, researchers measured allele diversity at the major histocompatibility complex (MHC), involved in the recognition of parasite-specific antigens. After one generation, diversity of MHC alleles was higher at the lake environment (which bears a broader range of parasites than the river environment), thus providing lake sticklebacks the advantage of fighting a more diverse set of parasites (Eizaguirre *et al*. 2012). In a similar experiment, researchers measured selection on natural allelic variants of the (*Eda*) locus, known to control adaptive differences in armour plates in sticklebacks (Colosimo *et al*. 2005). By transplanting marine sticklebacks harbouring both the low-plate and high-plate alleles of *Eda* into freshwater ponds and studying genotype frequency variations in one generation, researchers found that the low-plate allele was positively selected once lateral plates developed, likely because it provides a growth advantage in freshwater environments. However, the same allele was negatively selected before the plates were formed, indicating that either the *Eda* gene affects additional unmeasured traits under selection, or that tightly linked loci also have effects on fitness (Barrett & Schluter 2008). This last observation suggests that, when possible, tests of selection should be performed genome-wide using next-generation sequencing techniques in order to generate a high density of markers. This resolution can permit not only to confirm/detect selection signatures on the adaptive gene itself but also to detect the loci controlling unmeasured traits with fitness effects, and determine whether they are shaped by the same pleiotropic gene or by multiple linked loci (Barrett & Hoekstra 2011). To date, only a handful of studies have explored the genomic consequences of contemporary selection in the field using whole-genome data. In one study, researchers transplanted stick insects to native and novel host plants and measured allele frequency changes within a generation at genome-wide level (Gompert *et al*. 2014). In another study, patterns of genome-wide selection in purple sea urchins were evaluated under different ocean acidification levels (Pespeni *et al*. 2013). Both studies detect changes in allele frequencies driven by selection at multiple loci across the genome. However, as there was no previous knowledge of gene(s) underlying the formation of the adaptive traits directly affecting fitness, these results do not directly lead to conclusions about how selection affects allelic variance in a causal gene and the possible explanations for observing selection in non-causal regions of the genome (i.e. pleiotropy, indirect selection, linkage).

In addition, the combination of genomic data with selection experiments also gives the opportunity to evaluate the role of epistasis in adaptation. This has been applied to the study of laboratory adaptation using yeast, bacteria and viruses. These laboratory-based studies show a global pattern of diminishing returns epistasis (i.e. where the more mutations that accumulate, the weaker their fitness effect), which impedes the rate of ongoing adaptation relative to a null model of independent mutational effects (Chou *et al*. 2011; Khan *et al*. 2011; Kryazhimskiy *et al*. 2014). As the evolutionary patterns observed in small laboratory populations may not be the same as those contributing to natural evolution, the confirmation of these results still needs to be carried out in natural populations in order to determine the genomic effect of epistasis and its overall contribution to natural adaptation.

## Conclusions

Recent research has led to a remarkable growth in our understanding of the molecular basis of adaptive evolution (Table 1). Altogether, these studies have provided important insights into the genetic basis of adaptations and also the methodological approaches needed to answer this evolutionary problem. Nonetheless, it has become clear that the characterization of the genes underlying adaptive traits is not an easy task because factors such as demography, epistasis and pleiotropy can introduce confounding effects that will complicate any clear genetic signal. Also, methodological bias can mislead findings by pointing to large-effect loci and missing the detection of genes with small effect, thus complicating the description of ‘all’ important variants contributing to natural adaptation. Still, the search for the loci of evolution can benefit from following an organized and complementary methodology. First, it is necessary to corroborate that a trait affects fitness in the field and is in fact adaptive. Then, the region(s) of the genome in which genotypes are correlated with adaptive phenotypes should be defined either with classical genetic tools or applying new genomic approaches. Next, when DNA polymorphism associated with phenotype in the candidate genes does not occur in protein-coding regions, the expression pattern of such genes must be analysed for in order to test whether the trait has a *cis-* or *trans-*regulatory basis. Ultimately, functional experiments are required to prove that a gene or mutation is actually responsible for the phenotype observed. Once individual genes or SNPs have been identified, it is important to quantify their effect in the ‘trait value’ (i.e. how much variation in the phenotype is explained by the candidate SNPs/genes). Finally, the genetic variation in the genes shaping those adaptive traits should be evaluated in field selection experiments in order to establish a definite connection between genotype, phenotype and fitness.

A comprehensive review of the conclusions of such studies is beyond the scope of this article. However, some of the major findings include the following. First, the evolution of similar adaptive traits in different lineages commonly involves the action of the same genes (Colosimo *et al*. 2005; Nadeau & Jiggins 2010; Reed *et al*. 2011). Secondly, both *cis-*regulatory changes and coding changes contribute to adaptive variation (Mundy *et al*. 2004; Colosimo *et al*. 2005; Chan *et al*. 2010; Kunte *et al*. 2014). However, *cis-*regulatory changes may be more frequently involved in the evolution of morphological traits compared to physiological traits and in the evolution of morphological interspecific differences compared to the evolution of morphological intraspecific variation (Stern & Orgogozo 2008). Thirdly, the position of an adaptive gene in a regulatory network matters, as mutation in upstream patterning (input) genes is likely to affect the development of several body structures, while mutations in downstream (responsive) genes will influence the form of all incidences of the particular structure (Stern & Orgogozo 2008). However, *cis-*regulatory mutations in input/output genes provide great precision in evolutionary change with minimal pleiotropic effects (if any) (Stern & Orgogozo 2008; Gompel & Prud'homme 2009). Fourthly, adaptations can evolve from standing genetic variation, *de novo* mutations and adaptive introgression (Hermisson & Pennings 2005; Feldman, Brodie & Pfrender 2009; Hedrick 2013). Finally, most of the adaptations reported to date seem to arise through few initial mutations of major effect followed by many small effect mutations on minor genes (Orr 2005; Rockman 2011; Olson-Manning, Wagner & Mitchell-Olds 2012). Nonetheless, this last observation may be tainted by our experimental bias towards detecting large-effect alleles, so there is a likely ascertainment bias in the literature (Rockman 2011).

The search for the loci of evolution will be surely fuelled by the continuous increase in genomic and transcriptomics resources in natural populations, along with the development of novel methodologies applicable to such organisms. This offers exciting opportunities for testing new predictions and understanding how evolution proceeds.

## Data accessibility

This manuscript does not use data.
